# Healthcare access and adverse family impact among U.S. children ages 0–5 years by prematurity status

**DOI:** 10.1186/s12887-020-02058-0

**Published:** 2020-04-17

**Authors:** Olivia J. Lindly, Morgan K. Crossman, Amy M. Shui, Dennis Z. Kuo, Kristen M. Earl, Amber R. Kleven, James M. Perrin, Karen A. Kuhlthau

**Affiliations:** 1 Department of Health Sciences, Northern Arizona University, 1100 S. Beaver Street, Room 488, Flagstaff, AZ 86011 USA; 2 Building Bright Futures, Williston, Vermont USA; 3 Massachusetts General Hospital Biostatistics Center, Boston, MA USA; 4 Department of Pediatrics, University at Buffalo, Buffalo, New York USA; 5 Division of General Academic Pediatrics, Massachusetts General Hospital, Boston, MA USA; 6 Department of Pediatrics, Harvard Medical School, Boston, MA USA

**Keywords:** Prematurity, Low Birthweight, Early childhood, Healthcare access, Adverse family impact

## Abstract

**Background:**

Many children and their families are affected by premature birth. Yet, little is known about their healthcare access and adverse family impact during early childhood. This study aimed to (1) examine differences in healthcare access and adverse family impact among young children by prematurity status and (2) determine associations of healthcare access with adverse family impact among young children born prematurely.

**Methods:**

This was a secondary analysis of cross-sectional 2016 and 2017 National Survey of Children’s Health data. The sample included 19,482 U.S. children ages 0–5 years including 242 very low birthweight (VLBW) and 2205 low birthweight and/or preterm (LBW/PTB) children. Prematurity status was defined by VLBW (i.e., < 1500 g at birth) and LBW/PTB (i.e., 1500–2499 g at birth and/or born at < 37 weeks with or without LBW). Healthcare access measures were adequate health insurance, access to medical home, and developmental screening receipt. Adverse family impact measures were ≥ $1000 in annual out-of-pocket medical costs, having a parent cut-back or stop work, parental aggravation, maternal health not excellent, and paternal health not excellent. The relative risk of each healthcare access and adverse family impact measure was computed by prematurity status. Propensity weighted models were fit to estimate the average treatment effect of each healthcare access measure on each adverse family impact measure among children born prematurely (i.e., VLBW or LBW/PTB).

**Results:**

Bivariate analysis results showed that VLBW and/or LBW/PTB children generally fared worse than other children in terms of medical home, having a parent cut-back or stop working, parental aggravation, and paternal health. Multivariable analysis results only showed, however, that VLBW children had a significantly higher risk than other children of having a parent cut-back or stop work. Adequate health insurance and medical home were each associated with reduced adjusted relative risk of ≥$1000 in annual out-of-pocket costs, having a parent cut-back or stop work, and parental aggravation among children born prematurely.

**Conclusions:**

This study’s findings demonstrate better healthcare access is associated with reduced adverse family impact among U.S. children ages 0–5 years born prematurely. Population health initiatives should target children born prematurely and their families.

## Background

Many U.S. children are affected by preterm birth (gestational age < 37 weeks) and low birthweight (< 2500 g) in terms of their development and health across the life span [1–3]. Children born prematurely (i.e., preterm and/or low birthweight) are at higher risk than other children for chronic health conditions (e.g., cerebral palsy, developmental delay) [4–6] and challenges with language acquisition [7, 8], cognitive development and executive function [9, 10], and social and emotional development [11]. Children born prematurely also use more health services and incur greater healthcare costs than other children [12, 13], especially during the early childhood period when children are ages 0–5 years [14]. Poor child health, high service needs, and substantial costs may all contribute to adverse employment outcomes, stress, and poor mental health (e.g., depression) among parents of children born prematurely [15–17]. Still, knowledge is limited regarding the range of adverse family impacts—both financial and health related—experienced in early childhood among U.S. children born prematurely.

Easy access to quality pediatric healthcare may allay adverse family impacts for certain subgroups of children with special health care needs (e.g., those with autism spectrum disorder or attention deficit/hyperactivity disorder) [18–22]. For example, adequate health insurance coverage for children facilitates access to high quality healthcare including care delivered in a family-centered medical home [23, 24]. Care delivered in a family-centered medical home (medical home) is further related to developmental screening receipt among children [25]. Easy access to high quality healthcare (hereinafter referred to as healthcare access) may, in turn, reduce adverse family impact by providing the financial means and health services that children and their families need to thrive. Yet, U.S. children born prematurely are less likely than other children to have a medical home [26], and lacking a medical home is linked to poorer receipt of prescribed health services for children born prematurely [27]. Little research has, however, examined relationships between healthcare access and adverse family impact during early childhood for children born prematurely. Early childhood is a critical period for development and a time when families of children born prematurely may experience the greatest financial and health-related impact [13, 14, 28], therefore, warranting greater study.

**Fig. 1:**
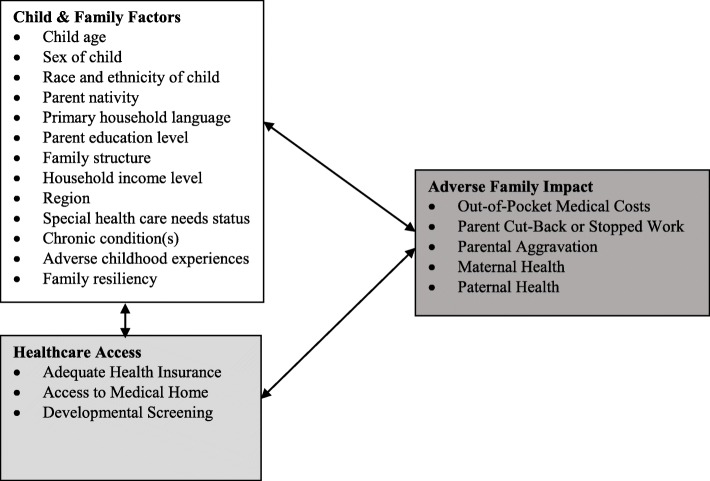
Conceptual Model of Relationships between Child & Family Factors, Healthcare Access, and Adverse Family Impact among U.S. Children ages 0–5 years Born Prematurely

To generate new knowledge regarding healthcare access and adverse family impact among young children according to prematurity status, we aimed to examine differences in healthcare access and adverse family impact among U.S. children ages 0–5 years by prematurity status and determine associations of healthcare access with adverse family impact among U.S. children ages 0–5 years born prematurely. Based on prior research examining healthcare access and adverse family impacts including parental health-related quality of life among children born prematurely or with other special health care needs [17, 18, 29–33], we hypothesized that young children born prematurely (i.e., very low birthweight or low birthweight and/or preterm) would have higher risk than other children of poor healthcare access (e.g., access to medical home) and adverse family impact (e.g., parent needing to cut-back or stop work, parental aggravation) than other families. We also hypothesized that healthcare access (e.g., adequate health insurance) would be associated with reduced risk of adverse family impact among young children born prematurely. This hypothesis stems from past research demonstrating that healthcare access is associated with reduced risk of adverse family impact for certain subgroups of children with special health care needs such as those with autism spectrum disorder [18, 20, 29]. In addition, because the socio-emotional health of children and their families is an essential aspect of the medical home model per *Bright Futures* guidelines [34], we hypothesized that linkages between healthcare access and adverse family impact such as parental aggravation and overall health were plausible among young children born prematurely. Figure 1 displays a conceptual model of the main constructs and indicators examined in this study.

## Methods

### Study design and data source

This study was a secondary analysis of publicly available, cross-sectional data that was combined from the 2016 and 2017 National Survey of Children’s Health (NSCH). The data analyzed for the current study are available through the U.S. Census Bureau at https://www.census.gov/programs-surveys/nsch/data.html. The NSCH is a parent-reported survey about healthcare access and quality, educational experiences, parent and family health, and child health for a nationally-representative sample of children ages 0–17 years. The NSCH is sponsored by the Maternal and Child Health Bureau of the Health Resources and Services Administration, part of the U.S. Department of Health and Human Services. The 2016 and 2017 NSCH were conducted by the U.S. Census Bureau using web- or mail-based survey administration, with a telephone questionnaire assistance option. Questionnaires were available in English or Spanish. The overall weighted response rates were as follows: 40.7% for the 2016 NSCH and 37.4% for the 2017 NSCH [35, 36]. Additional details about the NSCH methodology are available from the U.S. Census Bureau [37, 38].

Two parent advisors were continuously and regularly involved in the study’s conceptualization, design, and interpretation of results. Each parent advisor had a young child who was 2 to 3 years old that was born prematurely, and each advisor was involved on a family advisory committee for a neonatal intensive care unit (NICU) at a large academic medical center following their child’s discharge. The Institutional Review Board at Massachusetts General Hospital determined that this study was not human research and it was exempt from review.

### Participants

The full study sample included 19,482 U.S. children ages 0–5 years. We limited the study sample to children ages 0–5 years, because early childhood is a critical period for development and when children born prematurely and their families may experience the greatest adverse impact [13, 14, 28]. In the sample, 242 children were born very low birthweight (< 1500 g), 1236 children were born low birthweight (1500 to 2499 g), 969 children were born preterm but not low birthweight or very low birthweight, and 17,035 other children were not born very low birthweight, low birthweight, or preterm. Because children born preterm but not with low birthweight may be similarly prone to experience health risks as children born low birthweight (not very low birthweight) [4, 39], we combined children born low birthweight and children born preterm not low birthweight or very low birthweight (*n* = 2205) into one group (LBW/PTB) that was mutually exclusive from children born very low birthweight (VLBW) or other children. In both the 2016 and 2017 NSCH, parents were asked the following question to determine if children were born prematurely: “Was this child born more than 3 weeks before his or her due date?” To establish each child’s birthweight, parents were also asked: “How much did he or she weigh when born?” In alignment with the Centers for Disease Control and Prevention’s case definition [1], very low birthweight was defined as < 1500 g and low birthweight was defined as 1500 to 2499 g for this study.

### Measures

#### Healthcare access

Per past research about healthcare access and quality for child subgroups at high risk of health disparities (e.g., children with special health care needs, children with autism spectrum disorder) [29, 40–42], we used the following three healthcare access measures: adequate health insurance, access to a medical home, and developmental screening receipt. Adequate health insurance was a composite measure only assessed among children who were insured during the past 12-months. In the study sample, 635 children were uninsured. Adequate health insurance was determined by the following three subcomponents: health insurance benefits met the child’s needs (usually or always versus sometimes or never), coverage allowed the child to see needed providers (usually or always versus sometimes or never), and the child’s out-of-pocket health care expenses were reasonable (usually or always versus sometimes or never). To qualify as having adequate health insurance, children had usually or always on all three subcomponents. Access to a medical home was also a composite measure based on 16 items about the following five subcomponents of care in the past 12-months: child had a personal doctor or nurse, usual source for sick care, family-centered care (e.g., doctors spent enough time with the child, doctors showed sensitivity to family values and customs), no problems getting needed referrals, and effective care coordination when needed (e.g., got all needed help with care coordination, satisfaction with communication among child’s doctor and other health care providers). To qualify as having a medical home, children needed to have had a personal doctor or nurse, usual source for sick care, and family-centered care. To have been considered as having a medical home, children additionally must have had no problems getting needed referrals and effective care coordination (if they reported needing these services). Additional documentation about this medical home measure is provided elsewhere [43]. Developmental screening receipt was assessed with a 3-item measure previously validated using NSCH data [44]. The developmental screening measure was only assessed for children who were ages 9 to 35 months, in alignment with national screening guidelines [45]. Children were considered to have had developmental screening if their parent indicated a doctor or other health care provider had given them or another caregiver a questionnaire about specific concerns or observations they had about their child’s development, communication, or social behaviors and if this questionnaire had two age-specific content areas regarding language development and social behavior in the past 12-months.

#### Adverse family impact

We used five adverse family impact measures, which have been commonly used in relevant, past research [18, 20, 29]. Two of these measures were related to family financial and/or employment impacts including if the family spent $1000 or more on out-of-pocket medical expenses for the child during the past 12-months and if a parent or other family member cut down on hours working or stopped working because of the child’s health or health condition(s) during the past 12-months. Parental aggravation was a previously used composite measure derived from the following three items: parent felt the child is difficult to care for, parent felt that the child does things that bother them, and parent felt angry with the child [18]. All of the parental aggravation items were assessed for the past month and included a five-point response scale (never, rarely, sometimes, usually, always). Parents were defined as having often experienced parental aggravation during the past month if they indicated usually or always for any of the three measure items. Overall maternal and paternal health status not being excellent were similarly measured using two items: one item about the mother’s or father’s overall physical health status and one item about the mother’s or father’s overall mental health status. Each item was rated on a five-point scale (poor, fair, good, very good, excellent). Maternal and paternal health were both considered to be not excellent, if either physical or mental health status was reported to be poor, fair, good, or very good.

#### Covariates

We selected child and family characteristics as covariates that have established linkages with prematurity status, healthcare access, and/or adverse family impact and were available in the 2016 and 2017 NSCH [25, 27, 46, 47]. Covariates included the child’s age (years), sex (male or female), race and ethnicity (white and non-Hispanic, Hispanic, black and non-Hispanic, other race and non-Hispanic), parent’s nativity (born in the U.S. or not born in the U.S.), primary household language (English or Spanish/other language), highest parent education level (high school or less versus more than high school), family structure (two married parents, two unmarried parents, single mother, other family structure), household income level defined according to the family poverty ratio, health insurance coverage (private only, public only, private and public, uninsured or unspecified), and region of residence (Northeast, Midwest, South, West). In addition, the child’s special health care needs status was assessed by the Children with Special Health Care Needs (CSHCN) Screener [48]. Other covariates included current presence of one or more of 27 chronic conditions (e.g., asthma, developmental delay, speech and language disorder), number of adverse childhood experiences (e.g., parent divorced or separated, parent died), and family resiliency (i.e., family talks together about what to do when facing a problem, works together to solve a problem, knows the family has strengths to draw on when the family faces a problem, and stays hopeful even in difficult times when the family faces problems).

### Statistical analysis

We first compared characteristics of U.S. children ages 0–5 years by prematurity status using chi-square tests, as well as by using multinomial logistic regression for categorical variables and linear regression for continuous age. Both unadjusted and adjusted differences in healthcare access and adverse family impact by prematurity status were examined by estimating relative risk. All covariates that differed by prematurity status at a *p* < .10 level were included in the multivariable regression models used to compute adjusted differences in healthcare access and adverse family impact.

Given differences in healthcare access and adverse family impact by prematurity status and the study’s focus, we examined associations of healthcare access with adverse family impact only among children born prematurely (VLBW and PTB/LBW combined). Propensity score weighting was used to estimate the average treatment effect of each healthcare access indicator in relationship to each adverse family impact. We employed the propensity score weighting with subclassification approach recommended by DuGoff and colleagues when applying propensity score methods in using complex survey data such as that from the NSCH [49]. To compute propensity score weights, we initially included the following variables that were associated with ≥ 1 of the adverse family impact variables: age, sex of child, race/ethnicity, family structure, insurance status/type, region, VLBW status, CSHCN status, comorbid condition(s), ACE(s), family resilience, and the survey weights that the NCHS specified. We then assessed propensity score balance by evaluating the standardized differences of each covariate for each of the three healthcare access variables (adequate health insurance, medical home, developmental screening). Covariates were removed if the absolute value of the standardized difference was ≥ 0.10, and propensity scores were re-estimated with the remaining covariates. Different covariates were removed for models with each of the three healthcare access variables. Family structure, insurance status/type, CSHCN status, chronic condition(s), and family resilience were removed for adequate health insurance. Race/ethnicity, family structure, insurance status/type, CSHCN status, chronic condition(s), ACE(s), and family resilience were removed for medical home. Sex of child, race/ethnicity, family structure, insurance status/type, region, VLBW status, CSHCN status, and chronic condition(s) were removed for developmental screening. Doubly-robust estimators of causal effects and inverse probability of treatment weighting were used to weight the treatment (e.g., adequate health insurance) and comparison (e.g., no adequate health insurance) samples by the propensity scores for each adverse family impact variable. Standardized differences were again evaluated in the weighted samples, and the propensity score weights were multiplied by the survey weight to create a new weight used in fitting the weighted multivariable regression models. These relative risk models, with adverse family impact as the dependent variable and healthcare access as the main independent variable of interest, included the set of covariates that were initially considered for each propensity score and also adjusted for parent nativity, household language, and household income level (i.e., doubly-robust estimation). Family structure was omitted from the maternal and paternal health models due to possible collinearity with the dependent variable.

To better understand the healthcare access subcomponents contributing most to statistically significant associations with certain adverse family impacts, we additionally performed post-hoc bivariate and multivariable analyses to examine associations between adequate health insurance and medical home subcomponents and three adverse family impacts (out-of-pocket costs, parent cut-back or stopped work, parental aggravation) among children born prematurely. For these analyses, relative risk and 95% confidence intervals were estimated. Multivariable regression models included the same set of covariates initially used to examine differences in healthcare access and adverse family impact by prematurity status.

All analyses incorporated weighting to produce nationally representative estimates [38]. Weights were adjusted for multi-year analysis [50]. Family poverty ratio was analyzed in a multiple imputation framework [51]. We used a conventional alpha level of .05 to determine statistical significance. Given potential bias due to multiple comparisons made in the multivariable models, we additionally provided a Bonferroni-adjusted significance threshold to compare *p*-values against in relevant results tables. All analyses were performed in Stata version 15 [52].

## Results

As shown in Table 1, significant differences were found by prematurity status for race and ethnicity, household income level, health insurance coverage, special health care needs status, and current presence of one or more chronic health condition(s). Further pairwise comparison results showed that relative to other children: VLBW and LBW/PTB children were each more likely to be black and non-Hispanic versus white and non-Hispanic (RR = 2.39, 95% CI: 1.31–4.37, *p* = 0.005 and RR = 1.72, 95% CI: 1.26–2.35, *p* = 0.001, respectively), LBW/PTB children were more likely to be Hispanic versus white and non-Hispanic (RR = 1.96, 95% CI: 1.43–2.68, *p* < 0.001), VLBW and LBW/PTB children were each more likely to have public insurance coverage only (RR = 2.20, 95% CI: 1.22–3.96, *p* = 0.009 and RR = 1.37, 95% CI: 1.07–1.75, *p* = 0.013, respectively), children born VLBW and LBW/PTB were each more likely to have special health care needs (RR = 5.87, 95% CI: 3.39–10.18, *p* < 0.001 and RR = 1.67, 95% CI: 1.29–2.16, *p* < 0.001, respectively), and VLBW and LBW/PTB children were each more likely to have one or more chronic health condition(s) (RR = 2.36, 95% CI: 1.38–4.04, *p* = 0.002 and RR = 1.38, 95% CI: 1.10–1.73, *p* = 0.006, respectively). In terms of the individual chronic health conditions assessed in the NSCH, children born prematurely (i.e., VLBW or LBW/PTB) were most likely to have allergies, like other young children in the study sample. Developmental delay, speech and language disorders, and asthma were the next most frequent chronic conditions among children born prematurely.

**Table 1:** Characteristics of U.S. Children ages 0–5 years, by Prematurity Status (*n* = 19,482)

	Very Low Birthweight (*n* = 242)	Low Birthweight and/or Preterm (*n* = 2205)	Other Children (*n* = 17,035)	*p*-value
*Estimated Number* (%)	302,945 (1.4%)	293,8274 (13.3%)	18,818,572 (85.3%)	⏤
**Age, years**				0.58
*M* (*SD*)	2.3 (1.5)	2.5 (1.4)	2.5 (1.6)	
**Sex**				0.66
Male (*n* = 10,050)	49.2%	48.7%	51.2%	
Female (*n* = 9432)	50.8%	51.3%	48.8%	
**Race & Ethnicity**				< 0.001
White, non-Hispanic (*n* = 13,772)	39.2%	41.8%	55.9%	
Hispanic (*n* = 2086)	21.7%	31.3%	21.4%	
Black, non-Hispanic (*n* = 968)	17.8%	13.6%	10.6%	
Other race, non-Hispanic (*n* = 2656)	21.3%	13.2%	12.2%	
**Nativity**				0.17
Parent born in the U.S. *(n =* 15,362*)*	64.1%	70.7%	75.7%	
Parent not born in the U.S. *(n* = 3252*)*	35.9%	29.3%	24.3%	
**Primary Household Language**				0.09
English *(n =* 18,037*)*	79.1%	80.1%	87.1%	
Spanish or other language *(n =* 1343*)*	20.9%	19.9%	12.9%	
**Highest Parent Education Level**				0.16
High school or less (*n* = 2154)	29.1%	27.5%	22.3%	
More than high school (*n* = 17,230)	70.9%	72.5%	77.7%	
**Family Structure**				0.32
Two parents married (*n* = 15,306)	59.7%	65.2%	70.8%	
Two parents unmarried (*n* = 1494)	19.7%	11.4%	10.5%	
Single mother (*n* = 1723)	9.6%	14.1%	12.2%	
Other family structure (*n* = 815)	11.1%	9.3%	6.5%	
**Household Income Level**^**a**^				0.021
0–99% FPL (*n* = 1930)	24.2%	25.1%	18.7%	
100–199% FPL (*n* = 2584)	18.4%	22.6%	21.1%	
200–399% FPL (*n* = 6301)	29.1%	25.9%	28.5%	
≥ 400% FPL (*n* = 8257)	28.3%	26.4%	31.6%	
**Health Insurance Coverage**				0.019
Private health insurance only (*n* = 14,177)	41.2%	52.8%	59.1%	
Private and public health insurance (*n* = 703)	8.9%	4.2%	4.1%	
Public health insurance only (*n* = 3831)	47.6%	38.0%	31.1%	
Uninsured or unspecified insurance type (*n* = 679)	2.4%	5.0%	5.7%	
**Region**				0.21
Northeast (*n* = 3376)	20.4%	16.1%	16.4%	
Midwest (*n* = 5136)	17.3%	18.1%	22.0%	
South (*n* = 5919)	44.6%	39.4%	36.5%	
West (*n* = 5051)	17.7%	26.3%	25.0%	
**Children with Special Health Care Needs Status**				< 0.001
No (*n* = 17,210)	62.8%	85.6%	90.9%	
Yes (*n* = 2272)	37.2%	14.4%	9.2%	
**≥1 Current Chronic Health Condition(s)**				< 0.001
No (*n* = 13,864)	58.0%	70.3%	76.5%	
Yes (*n* = 4739)	42.0%	29.7%	23.5%	
**Adverse Childhood Experience(s)**^**b**^				0.16
None (*n* = 13,402)	47.1%	63.9%	65.9%	
1 Adverse childhood experience (*n* = 3706)	43.5%	23.6%	23.1%	
≥ 2 Adverse childhood experiences (*n* = 1683)	9.3%	12.4%	11.0%	
**Family Resilience**^**c**^				0.67
Some/none of the time 0–1 items (*n* = 1022)	12.9%	6.5%	6.5%	
Most of the time 2–3 items (*n* = 1791)	10.1%	11.1%	8.8%	
All of the time to all 4 items (*n* = 16,669)	77.0%	82.5%	84.7%	

Data source: 2016 & 2017 National Survey of Children’s Health

*Abbreviations*: *FPL* federal poverty level, *U.S.* United States

^a^Weighted percentages were estimated from multiple imputation

^b^The following 9 adverse childhood experiences were assessed in the 2016 and 2017 NSCH: hard to get by on family’s income, parent or guardian divorced or separated, parent or guardian died, parent or guardian served time in jail, witnessed domestic violence, lived with anyone who was mentally ill, suicidal or severely depressed, lived with anyone who had a problem with alcohol or drugs, and treated or judged unfairly because of his/her race or ethnic group

^c^The following 4 indicators of family resilience were assessed in the 2016 and 2017 NSCH: talk together about what to do when the family faces a problem, work together to solve the problem when the family faces problems, know we have strengths to draw on when the family faces problems, and stay hopeful even in difficult times when the family faces problems

As shown in Table 2, bivariate analysis results demonstrated that LBW/PTB were less likely to have had a medical home compared to other children, and VLBW children were more likely than other children to have received developmental screening. Neither of these associations remained statistically significant, however, after adjusting for other factors. For adverse family impact, bivariate analysis results demonstrated that VLBW children had higher risk than other children of having a parent who cut-back and/or stopped work because of the child’s health condition, parental aggravation, and less than excellent paternal health. LBW/PTB children also had higher risk than other children of having a parent cut-back or stop work according to bivariate analysis results. Multivariable analysis results showed that only VLBW children had higher risk of having a parent cut-back or stop work compared to other children.

**Table 2:** Healthcare Access and Adverse Family Impact among U.S. Children ages 0–5 years, by Prematurity Status

	Very Low Birthweight	Low Birthweight and/or Preterm	Other Children
**Healthcare Access**			
Adequate Health Insurance	79.0%	73.6%	72.3%
RR (95% CI)	1.09 (0.98–1.22)	1.02 (0.96–1.08)	1.00
aRR (95% CI)	1.05 (0.95–1.16)	0.99 (0.93–1.05)	1.00
*p*-value	0.32	0.69	–
Medical Home	42.2%	42.6%	51.8%
RR (95% CI)	0.81 (0.59–1.12)	0.82 (0.72–0.94)	1.00
aRR (95% CI)	0.88 (0.66–1.17)	0.91 (0.80–1.03)	1.00
*p*-value	0.38	0.14	–
Developmental Screening	51.9%	37.7%	32.9%
RR (95% CI)	1.58 (1.03–2.42)	1.15 (0.91–1.44)	1.00
aRR (95% CI)	1.49 (0.96–2.32)	1.18 (0.96–1.44)	1.00
*p*-value	0.08	0.11	–
**Adverse Family Impact**			
≥ $1000 Out-of-Pocket Expenses	13.5%	13.2%	12.3%
RR (95% CI)	1.10 (0.67–1.81)	1.08 (0.83–1.39)	1.00
aRR (95% CI)	1.06 (0.69–1.64)	1.20 (0.94–1.54)	1.00
*p*-value	0.78	0.15	–
Parent Cut-back or Stopped Work	30.3%	8.6%	5.1%
RR (95% CI)	5.95 (3.59–9.86)	1.68 (1.19–2.38)	1.00
aRR (95% CI)	2.91 (1.86–4.56)	1.41 (0.98–2.02)	1.00
*p*-value	<.001	0.06	–
Parental Aggravation	9.3%	4.3%	3.1%
RR (95% CI)	3.02 (1.59–5.71)	1.39 (0.93–2.10)	1.00
aRR (95% CI)	1.41 (0.83–2.39)	1.14 (0.76–1.71)	1.00
*p*-value	0.20	0.54	–
Maternal Health Not Excellent	73.7%	76.8%	73.5%
RR (95% CI)	1.00 (0.86–1.18)	1.04 (0.98–1.11)	1.00
aRR (95% CI)	0.98 (0.83–1.15)	1.03 (0.97–1.10)	1.00
*p*-value	0.80	0.32	–
Paternal Health Not Excellent	81.5%	72.4%	71.0%
RR (95% CI)	1.15 (1.02–1.29)	1.02 (0.95–1.10)	1.00
aRR (95% CI)	1.12 (0.99–1.28)	1.01 (0.94–1.09)	1.00
*p*-value	0.08	0.71	–

*Note*. Each multivariable model included the following covariates in addition to prematurity status: child race and ethnicity, household language, household income, insurance coverage, children with special health care needs status, and one or more chronic condition(s). *P*-values are provided for the multivariable models that estimated adjusted relative risk ratios. For these models, the Bonferroni-adjusted significance threshold = 0.006

Data source: 2016 & 2017 National Survey of Children’s Health

*Abbreviations*: *aRR* adjusted relative risk, *CI* confidence interval, *RR* relative risk, *U.S.* United States

Propensity weighted multivariable regression model results showed that among U.S. children ages 0–5 years who were born prematurely: adequate health insurance and medical home were each associated with significantly lower risk of $1000 or more in annual, out-of-pocket medical expenses, having a parent who cut-back or stopped work, and parental aggravation (Table 3). Developmental screening receipt did not have a statistically significant association with any of the adverse family impacts. None of the healthcare access measures had statistically significant associations with less than excellent maternal or paternal health status.

**Table 3:** Associations of Healthcare Access with Adverse Family Impact among U.S. Children Born Prematurely (VLBW and LBW/PTB combined), ages 0–5 years

	Adverse Family Impact				
	≥$1000 Out-of-Pocket Expenses	Parent Cut-Back or Stopped Work	Parental Aggravation	Maternal Health Not Excellent	Paternal Health Not Excellent
Healthcare Access					
Adequate Health Insurance					
RR (95% CI)	0.21 (0.12–0.36)	0.38 (0.22–0.66)	0.37 (0.19–0.71)	0.96 (0.84–1.08)	0.97 (0.86–1.09)
*p*-value	<.001	0.001	0.003	0.48	0.62
aRR (95% CI)	0.25 (0.15–0.42)	0.26 (0.16–0.44)	0.44 (0.26–0.74)	0.92 (0.83–1.02)	0.95 (0.85–1.07)
*p*-value	<.001	<.001	0.002	0.13	0.39
Medical Home					
RR (95% CI)	0.48 (0.22–1.08)	0.22 (0.09–0.55)	0.27 (0.10–0.73)	1.12 (0.98–1.29)	1.13 (0.96–1.34)
*p*-value	0.08	0.001	0.010	0.09	0.14
aRR (95% CI)	0.54 (0.38–0.76)	0.34 (0.17–0.70)	0.32 (0.17–0.60)	1.04 (0.95–1.13)	1.01 (0.92–1.12)
*p*-value	<.001	0.004	<.001	0.41	0.78
Developmental Screening					
RR (95% CI)	1.00 (0.48–2.09)	2.29 (0.80–6.51)	1.26 (0.27–5.85)	1.08 (0.84–1.38)	1.12 (0.88–1.43)
*p*-value	0.997	0.12	0.77	0.56	0.37
aRR (95% CI)	0.66 (0.39–1.11)	1.13 (0.52–2.43)	0.69 (0.28–1.66)	1.04 (0.87–1.23)	1.20 (0.99–1.46)
*p*-value	0.12	0.76	0.40	0.70	0.056

*Note.* Propensity score weighting was used to estimate average treatment effect of adequate health insurance, access to medical home, or developmental screening receipt on each adverse family impact. Models included the following covariates: child age, sex, race and ethnicity, family structure, insurance status/type, region, VLBW status, CSHCN status, comorbid condition(s), ACEs, family resilience, parent nativity, household language, and household income. Family structure was omitted from maternal and paternal health models due to possible collinearity with the dependent variable. For these models, the Bonferroni-adjusted significance threshold = 0.003

Data source: 2016 & 2017 National Survey of Children’s Health

*Abbreviations*: *aRR* adjusted relative risk, *CI* confidence interval, *LBW* low birthweight, *PTB* preterm birth, *RR* relative risk, *U.S.* United States, *VLBW* very low birthweight

Post-hoc sensitivity analysis results showed that each adequate health insurance subcomponent (i.e., health insurance benefits always met child’s needs, coverage always allowed child to see their needed provider(s), out-of-pocket medical expenses were always reasonable) was associated with significantly lower adjusted risk of $1000 or more in annual, out-of-pocket medical expenses and having a who parent cut-back or stopped work among children born prematurely (Appendix). Only the adequate health insurance subcomponent of having coverage that always allowed the child to see needed providers was associated with significantly lower adjusted risk of parental aggravation. For the medical home subcomponents, effective care coordination was consistently associated with reduced adjusted risk of each of the three adverse family impacts examined. No problems getting needed referrals and family-centered care were each associated with significantly reduced adjusted risk of having a parent who cut-back or stopped work. Having a usual source of sick care was significantly associated with reduced adjusted risk of parental aggravation.

## Discussion

This study’s findings demonstrate that young children born prematurely may be at higher risk of poor healthcare access and adverse family impact relative to young children not born prematurely in the United States. Moreover, among young children born prematurely adequate health insurance and medical home were each associated with reduced risk of high out-of-pocket medical expenses, having a parent cut-back or stop work, and parental aggravation. Together, these findings highlight the importance of healthcare access in relationship to adverse family impact during the early childhood period for U.S. children born prematurely.

Study findings regarding differences in healthcare access and adverse family impact by prematurity status are fairly consistent with past research. In line with our hypothesis about healthcare access, LBW/PTB children were less likely than other children to have a medical home; however, this difference did not remain statistically significant in the multivariable analysis results. Still, less than half of children born prematurely in this study had a medical home, and past research using 2011/12 NSCH data has similarly shown that children ages 0–3 years born prematurely (i.e., VLBW and LBW) are less likely to have a medical home compared to other children [27]. For these reasons, further efforts are needed to ensure children born prematurely have a medical home after NICU discharge. Post-discharge plans along with care coordination and co-management that connects parents of children born prematurely to medical homes at or affiliated with a larger integrated healthcare system (e.g., healthcare systems providing pediatric therapy services and adult health services) may make medical home access more logistically feasible for parents needing specialty and therapy services for their child, as well as healthcare for themselves [53, 54]. Past qualitative inquiry conducted to understand parent and healthcare provider experiences around the time of NICU discharge may provide a foundation for future efforts to better facilitate medical home access for children born prematurely [55–58].

Contrary to our hypothesis regarding healthcare access, VLBW children were more likely than other children to receive developmental screening; however, this difference did not remain statistically significant in the multivariable analysis results. Because VLBW children are at greater risk of developmental disability and special health care needs relative to children born LWB [4], developmental surveillance and screening may, in practice, happen more frequently for this subgroup. In addition, certain states have started to specify very low birthweight as a criterion for early intervention eligibility [59, 60], plausibly increasing awareness among both healthcare providers, educators, and parents regarding the importance of developmental screening for this subgroup. Given the benefits that developmental screening and early intervention access may have for young children born prematurely [61], continued efforts are warranted to increase developmental screening for children born prematurely. Existing initiatives intended to promote developmental screening and access to related services (e.g., early intervention, early childhood special education services) may consider explicitly raising public awareness about the elevated risk of developmental disability for children born prematurely. Efforts to educate and/or follow-up with parents about developmental milestones and screening around the time of NICU discharge may also help to bolster screening rates and provide additional opportunities for parents to access services for themselves (e.g., referral to counseling for depression).

As expected, children born prematurely—particularly those born VLBW— were at high risk of having a parent cut-back or stop work. Strikingly, nearly one-third of parents whose children were ages 0–5 years and were born VLBW reported that either they or another family member had cut-back or stopped work because of the child’s health condition. Parents of young children born prematurely may need to cut-back or stop work given the higher volume of healthcare services that their children are likely to require [13, 14], as well as difficulty accessing early child care services. Because U.S. children born prematurely are disproportionately born to families who are low income and/or of color [3], it is also important to ensure that healthcare policy and systems address programs and factors supporting socioeconomic family financial and employment circumstances including barriers that families with greater disadvantage are likely to encounter. From the time infants are admitted to the NICU, team-based care that involves social workers and/or other health professionals can utilize structured assessments, facilitated enrollment, and system navigation for family income and support programs (e.g., temporary assistance for needy families; women, infants, and children program; supplemental nutrition assistance program; respite care). Ongoing supports should span the hospital-to-home transition and be incorporated within the medical home settings as well. Embedding such services within bundled payment programs may also make such services more financially viable for healthcare systems. Family Medical Leave Act expansion across states may also be considered as a larger scale policy shift that could reduce financial and employment burden for families of young children born prematurely.

In relationship to our second central hypothesis, adequate health insurance and medical home were each associated with reduced risk of high out-of-pocket medical expenses, having a parent cut-back or stop work, and parental aggravation. From our sensitivity analysis results, all adequate health insurance components were salient in terms of their statistically significant associations with these three adverse family impacts. For the medical home subcomponents, effective care coordination had the strongest association with these adverse family impacts followed by no problems getting needed referrals and family-centered care. Collectively, this pattern of results suggests that medical home implementation and comprehensive care standards can improve outcomes for children born prematurely and their families. Yet, healthcare access was not significantly associated with overall maternal or paternal health status suggesting that other factors may be at play and need to be addressed to promote parental health for young children born prematurely. It is also possible that relatively poor maternal and/or paternal health is a risk factor for prematurity or that prematurity itself is a risk factor for suboptimal parental health. In either case, access to care for children born prematurely and their parents remains paramount to promoting health. Here again, quantitative and qualitative inquiry building on past research that has involved parents of children born prematurely may be needed to better understand pathways to health for parents of children born prematurely and by the extent of prematurity [56–58].

### Limitations and strengths

This study’s findings should be interpreted with its main limitations in mind. First, this study was a secondary analysis of cross-sectional data so we cannot understand longitudinal pathways between healthcare access and adverse family impact during the early childhood period among children born prematurely. Nonetheless, this study utilized recent and nationally-representative data on young children born prematurely. We were also limited in our categorization of prematurity status by the survey items used. That is, we do not know the exact gestational age of children who were reported to be born preterm. Similarly, both preterm and birthweight status were assessed based on parent report, which may have been inaccurate especially for children who were older. Still, by using 2 years of NSCH data, we were able to differentiate children born very low birthweight. In addition, not all aspects of healthcare access and adverse family impact (e.g., trauma experienced during pregnancy) were accounted for by the measures used in this study. For instance, we do not know if families did not change jobs to avoid losing their family medical leave eligibility or parents experienced post-traumatic stress disorder related to their child’s premature birth and/or their own health complications from the birth. Relatedly, we do not know if families were part of a NICU follow-up program that could have potentially influenced their child’s health insurance adequacy and medical home status.

## Conclusions

This study used nationally-representative data to demonstrate differences in healthcare access and adverse family impact by prematurity status among children ages 0–5 years. Findings show better healthcare access is associated with reduced adverse family impact in early childhood among U.S. children born prematurely. Children born prematurely and their families are susceptible to poor health outcomes and should be targeted in population health initiatives. Policy-, practice-, and family-level interventions exist but require further work to improve health for this vulnerable population.

## Data Availability

This study was a secondary analysis of publicly available, cross-sectional data that was combined from the 2016 and 2017 National Survey of Children’s Health (NSCH). The data analyzed for the current study are available through the U.S. Census Bureau at https://www.census.gov/programs-surveys/nsch/data.html.

## Appendix

Sensitivity Analysis Results: Associations of Adequate Health Insurance and Medical Home Subcomponents with Adverse Family Impacts among U.S. Children Born Prematurely (VLBW and LBW/PTB combined), ages 0–5 years.

**Table:**

	Adverse Family Impact		
	≥$1000 Annual Out-of-Pocket Expenses	Parent Cut-back or Stopped Work	Parental Aggravation
Healthcare Access			
**Adequate Health Insurance Subcomponents**			
Health insurance benefits always met child’s needs			
*%*	8.7%	4.2%	2.7%
RR (95% CI)	0.40 (0.34–0.46)	0.40 (0.31–0.51)	0.51 (0.37–0.69)
aRR (95% CI)	0.48 (0.42–0.55)	0.47 (0.36–0.61)	0.62 (0.45–0.85)
*p*-value	<.001	<.001	.003
Coverage always allowed child to see needed providers			
*%*	10.5%	4.9%	2.8%
RR (95% CI)	0.46 (0.39–0.54)	0.41 (0.32–0.52)	0.44 (0.32–0.60)
aRR (95% CI)	0.53 (0.46–0.61)	0.49 (0.38–0.63)	0.55 (0.40–0.74)
*p*-value	<.001	<.001	<.001
Always had reasonable out-of-pocket expenses			
*%*	5.2%	2.5%	2.2%
RR (95% CI)	0.20 (0.16–0.26)	0.32 (0.19–0.54)	0.61 (0.40–0.94)
aRR (95% CI)	0.21 (0.16–0.27)	0.39 (0.24–0.65)	0.81 (0.53–1.23)
*p*-value	<.001	<.001	.32
**Medical Home Subcomponents**			
Personal doctor or nurse			
%	13.2%	6.0%	3.5%
RR (95% CI)	1.39 (1.15–1.68)	1.03 (0.76–1.40)	1.21 (0.83–1.75)
aRR (95% CI)	1.06 (0.88–1.28)	0.89 (0.66–1.21)	1.02 (0.69–1.50)
*p*-value	.53	.45	.94
Usual source for sick care			
*%*	13.5%	5.8%	2.9%
RR (95% CI)	1.72 (1.28–2.32)	0.92 (0.64–1.32)	0.62 (0.41–0.94)
aRR (95% CI)	1.04 (0.78–1.40)	0.90 (0.62–1.30)	0.50 (0.35–0.73)
*p*-value	0.77	0.57	<.001
Family-centered care			
*%*	12.9%	5.2%	3.2%
RR (95% CI)	0.99 (0.77–1.26)	0.41 (0.31–0.54)	0.54 (0.37–0.78)
aRR (95% CI)	0.71 (0.57–0.90)	0.54 (0.40–0.72)	0.70 (0.47–1.03)
*p*-value	.004	<.001	.07
No problems getting referrals			
*%*	21.7%	12.7%	6.0%
RR (95% CI)	1.13 (0.84–1.52)	0.44 (0.32–0.62)	0.47 (0.31–0.73)
aRR (95% CI)	0.84 (0.66–1.08)	0.57 (0.41–0.79)	0.59 (0.37–0.93)
*p*-value	.17	.001	.023
Effective care coordination			
*%*	14.2%	5.3%	3.2%
RR (95% CI)	0.65 (0.55–0.79)	0.29 (0.22–0.38)	0.33 (0.23–0.48)
aRR (95% CI)	0.61 (0.52–0.72)	0.43 (0.32–0.57)	0.49 (0.34–0.71)
*p*-value	<.001	<.001	<.001

*Note*. Each multivariable model included the following covariates in addition to prematurity status: child race and ethnicity, household language, household income, insurance coverage, children with special health care needs status, and chronic condition(s). *P*-values are provided for the multivariable models that estimated adjusted relative risk ratios. For these models, the Bonferroni-adjusted significance threshold = 0.002

Data source: 2016 & 2017 National Survey of Children’s Health

*Abbreviations*: *aRR* adjusted relative risk, *CI* confidence interval, *LBW* low birthweight, *PTB* preterm birth, *RR* relative risk, *U.S.* United States, *VLBW* very low birthweight

## Abbreviations

CI: Confidence interval
CSHCN: Children with special health care needs
FPL: Family poverty ratio
NSCH: National Survey of Children’s Health
RR: Relative risk
